# Iron–sulfur clusters: from metals through mitochondria biogenesis to disease

**DOI:** 10.1007/s00775-018-1548-6

**Published:** 2018-03-06

**Authors:** Mauricio Cardenas-Rodriguez, Afroditi Chatzi, Kostas Tokatlidis

**Affiliations:** Institute of Molecular, Cell and Systems Biology, College of Medical, Veterinary and Life Sciences, University of Glasgow, Glasgow, G12 8QQ UK

**Keywords:** Metal, Cysteine, Iron–sulfur, Mitochondrial disease, Iron regulation

## Abstract

Iron–sulfur clusters are ubiquitous inorganic co-factors that contribute to a wide range of cell pathways including the maintenance of DNA integrity, regulation of gene expression and protein translation, energy production, and antiviral response. Specifically, the iron–sulfur cluster biogenesis pathways include several proteins dedicated to the maturation of apoproteins in different cell compartments. Given the complexity of the biogenesis process itself, the iron–sulfur research area constitutes a very challenging and interesting field with still many unaddressed questions. Mutations or malfunctions affecting the iron–sulfur biogenesis machinery have been linked with an increasing amount of disorders such as Friedreich’s ataxia and various cardiomyopathies. This review aims to recap the recent discoveries both in the yeast and human iron–sulfur cluster arena, covering recent discoveries from chemistry to disease.

## Introduction

Iron–sulfur clusters are metal prosthetic groups, synthesized and utilised in different cell compartments. They are present in nearly all organisms and required for a variety of protein biological functions, such as enzyme activity, protein regulation, and translation [1–6]. Fe–S clusters are considered to be among the most ancient catalysts, a concept that is supported by the elevated iron and sulfur levels in the environment and their unique characteristics, including the electron charge transfer activity and the formation into complexes [7, 8].

Despite their abundance, one of the many challenges which this field had to face early on was the detection of the clusters on proteins. The isolation of an intact complex of the protein with the cluster has been a difficult task, as the cluster can easily dissociate or change its redox state. These issues along with the lack of additional structural protein information were the cause for slow paced study of the clusters.

**Fig. 1:**
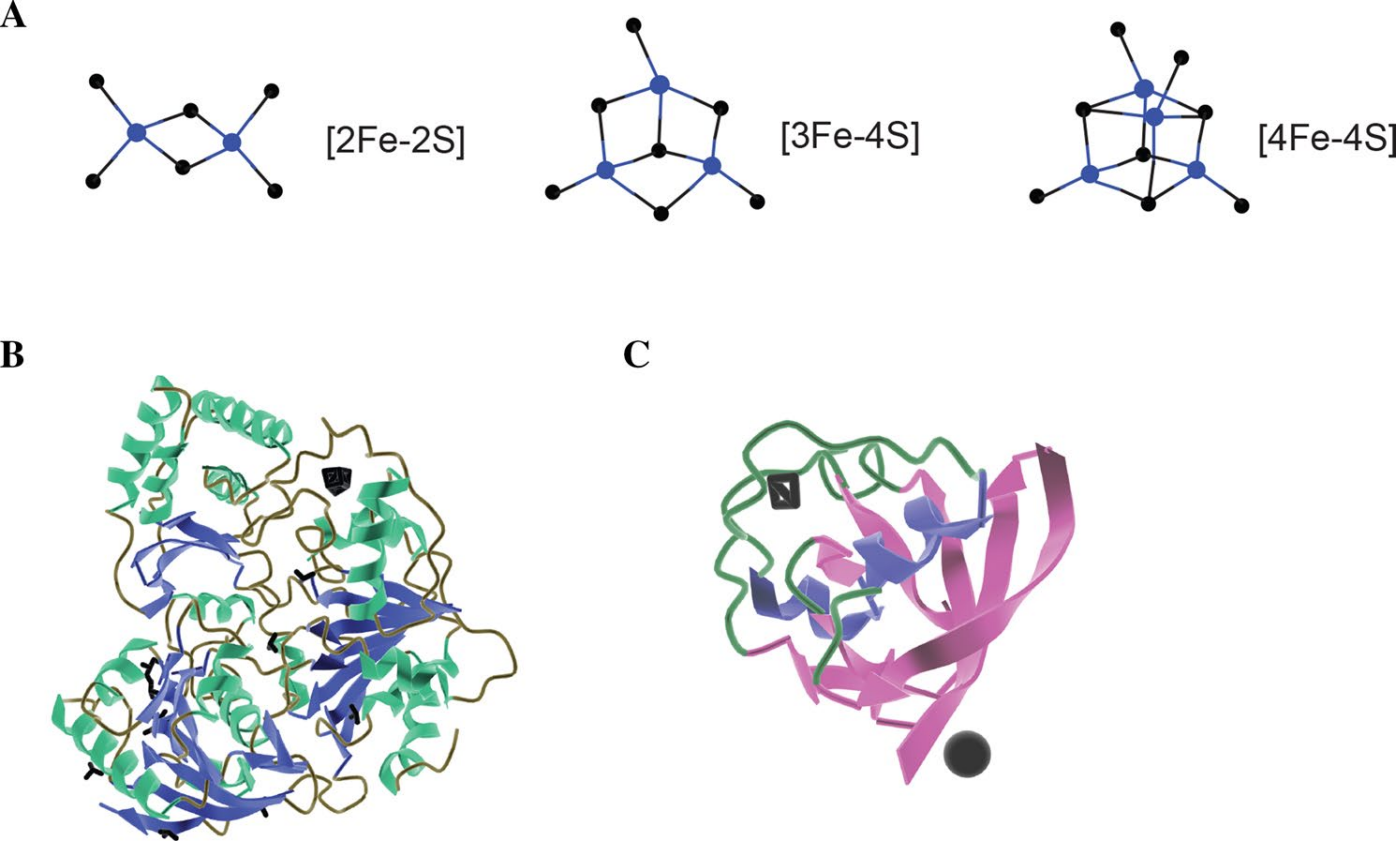
Iron–sulfur clusters and proteins. **a** Example structures of known and well-characterised Fe/S clusters. Blue; Fe, black; sulfur. **b** Crystal structure of XPD helicase (PDB ID 3CRV)  and **c** ferredoxin 2 (PDB ID 4ZHO) with a [4Fe–4S] and [2Fe–2S] clusters bound (black sticks)

Consequently, although Fe–S clusters have been studied for decades, many aspects of their synthesis, transfer, incorporation on proteins, and role have still to be understood [9–11]. Even though Fe–S clusters can be found in different chemical structures, all of them derive from iron ions and sulfide (Fig. 1). The simplest form that can be found in nature is [2Fe–2S] and duplication of this form results to the cubic cluster [4Fe–4S]. In addition, loss of one Fe ions of that form leads to the non-symmetrical [3Fe–4S] structure [10]. Finally, another form is composed of a cubane [4Fe–4S] together with a [4Fe–3S] cluster resulting in the [8Fe–7S] cluster [3, 12].

These inorganic moieties pose an excellent example of co-factors that are highly reactive and thus suitable for many catalytic reactions. However, this feature is the main cause of their susceptibility as they are also prone to oxidation that leads to the Fe–S cluster inactivation. Many enzymes, such as DNA-binding proteins, have traded over iron for zinc or other metals that are less sensitive to oxidation and thus less likely to cause toxicity. Specifically, it was originally believed that glycosylases were the only DNA-binding enzymes that utilise Fe–S clusters [2, 13]. However, in 2006, another DNA enzyme, the helicase XfPD (Rad3 in *Saccharomyces cerevisiae*) was shown to bind to an iron–sulfur cluster within the catalytic domain of the protein. This finding indicated a role for iron–sulfur clusters in sensing DNA disruption (Fig. 1) and led to discovering more DNA-binding proteins that use Fe–S clusters, including all DNA polymerases [14–16]. The role of Fe–S clusters in processing nucleic acids is still not fully understood, although they are indispensable for enzyme activity. The main question focuses on why these metal moieties are preferred over others, when the risk of toxicity or DNA damage by iron is so great. This is a critical research area that can link Fe–S cluster chemistry with protein function, DNA damage, and subsequently diseases.

In this review, we will first describe the steps for the Fe–S biogenesis in the model organism *Saccharomyces cerevisiae* (budding yeast) and expand onto the relevant mechanisms in mammalian cells covering the recent information on the transport and delivery of the clusters into apoproteins. Given the importance that mitochondria have on Fe–S cluster formation, we will discuss any putative links between that pathway and the organelle’s biogenesis. Finally, some of the main human disorders caused by defects in proteins underpinning the Fe–S biogenesis machinery will be discussed.

## Fe–S cluster pathways: from yeast to mammalian cells

For decades, it was presumed that Fe–S clusters are incorporated on apoproteins in a spontaneous fashion, a theory that was proved insufficient, since in vitro successful integration required toxic Fe levels. A more plausible theory was that there are specific pathways responsible for the biosynthesis of Fe–S for the final delivery to apoproteins. Later, studies on the fields of iron chemistry, metabolism, and oxidative stress provided the evidence that changed the perception of Fe–S biosynthesis [17–19]. One of the main breakthroughs was the investigation of the enzyme nitrogenase [20], that catalyses the reduction of dinitrogen, known as ‘nitrogen fixation’. Dennis Dean and colleagues performed genetic and biochemical studies on the Gram-negative facultative anaerobe *Klebsiella pneumoniae* bacterium identifying genes involved in the nitrogen fixation process. The comparison between the sequences of the nifUSV gene clusters from *K. pneumoniae* and *Azotobacter vinelandii* revealed identical organization of the genomes as well as a high degree of sequence homology [21]. The studies made on *A. vinelandii* were crucial to the discovery of Fe–S biogenesis mechanism as three operons dedicated to the Fe–S biogenesis were found, one of them being the *nif* operon, involved in the biogenesis of nitrogenase [22]. Since then, the iron–sulfur cluster synthesis and assembly pathways have begun to be described, in addition to the sensing iron-level mechanisms, providing a better understanding of the complicated chemistry of iron [23, 24]. Bacteria Fe–S cluster biogenesis machineries have been reviewed elsewhere [25, 26], and thus, are not the focus of this review. The data summarised below have been acquired from studies mainly in yeast and mammalian cell lines. Since the pathways are conserved, we only shortly refer to the yeast system and analyse, in more detail, the mammalian as a way to introduce the relevance and implication of this co-factor in a wide range of diseases.

## Fe–S clusters in *Saccharomyces cerevisiae*

Iron–sulfur cluster proteins can be found in most cell compartments from mitochondria to the nucleus. Due to their role complexity, the need for a simple model system to study these molecules was apparent. The yeast *S. cerevisiae* has been used in the past thoroughly to study complex biological processes as a simple, easy to grow eukaryotic model organism and provided the necessary platform for the investigation of the highly conserved Fe–S pathways.

**Fig. 2:**
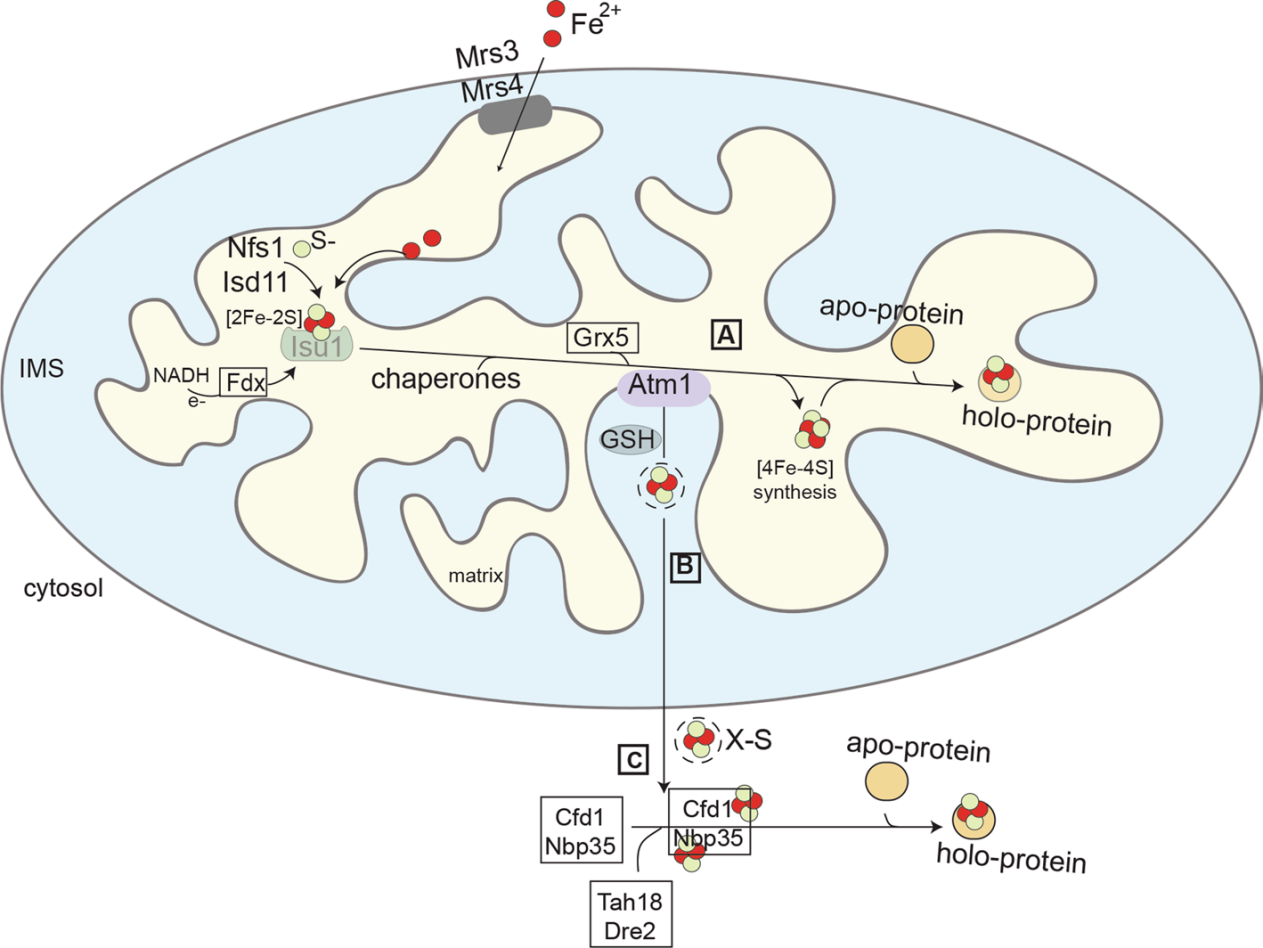
Fe/S cluster biogenesis. The process includes three different stages, two in mitochondria (A and B), and one in the cytosol (C). In the mitochondrial matrix, the ISC machinery (A) is responsible for the formation of the clusters and the maturation of the apoproteins. An unknown compound (X-S) is exported from the matrix (B) through the ISC export pathway. In the cytosol (C), the CIA machinery takes over for the incorporation of the clusters in the proteins

Free iron (as well as sulfides) can be toxic to the cell [27, 28], rendering its regulation, uptake, and assembly essential processes that should be controlled constantly. Three biosynthesis pathways have been described so far that contribute to that end; two at the mitochondrial level [iron–sulfur cluster (ISC)] and one at the cytosolic compartments [cytosolic iron–sulfur cluster assembly (CIA)] (Fig. 2) [2, 11, 29, 30]. In the mitochondrial matrix, the first stage of the ISC pathway comprises of the iron–sulfur cluster synthesis, with the extraction of sulfur from cysteine. This reaction is catalysed by the enzyme cysteine desulfurase, Nfs1. Next is the cluster assembly, a step that is accomplished on the scaffold protein Isu1 by a group of enzymes that utilise the available iron and sulfur, as shown in Fig. 2. The Fe–S cluster is subsequently released from the scaffold protein and transferred by chaperones to glutaredoxin 5 (Grx5). The cooperation between Grx5 and co-chaperones, Jac1 and Ssq1, leads to the final incorporation of the clusters onto the target apoproteins [31]. Apart from the simple cluster, the mitochondrial ISC machinery can also produce the more complex forms that are required for the function of several proteins in the organelle. The synthesis of a cubic cluster from the initially formed [2Fe–2S] requires a conversion reaction occurring later in the pathway. Alternatively, the [2Fe–2S] clusters follow the putative mitochondrial export route to be used by the CIA system.

The exported form of the cluster has been the subject of many studies over the years; and despite substantial progress in the field, the identity of the exported species remains speculative. Originally, it was shown that the export pathway involves three components, one membrane channel protein (Atm1), one sulfhydryl oxidase (Erv1), and one reducing factor [glutathione (GSH)]. The equivalent human homologues will be reviewed later. The most recent data show that the exported cluster could be stabilised by glutathione molecules [32, 33]. Specifically, crystal structures revealed that reduced glutathione (GSH) was associated with both the yeast and bacterial pore Atm1, whereas bacterial Atm1 could also bind to oxidised glutathione (GSSG). However, there are still no concrete data of transported conjugates of glutathione and iron–sulfur cluster, rendering the real nature compound elusive. In addition, there are studies that contradict the presence of an export pathway, based on the inconclusive evidence of an exported cluster from mitochondria and the cytosolic presence of the initial proteins involved in the cluster formation [34].

In the cytoplasm, a [2Fe–2S] cluster that is in turn bound by the two monothiol glutaredoxins, Grx3 and Grx4 [35]. The two proteins act as a homodimer coordinating the cluster with the assistance of two glutathione molecules [35, 36]. The Fe–S cluster can be further used for the biogenesis of cytosolic and nuclear Fe–S proteins. Specifically, the two proteins Tah18–Dre2 provide the necessary electron donors to deliver the assembly of the cluster on the first scaffold protein complex of the CIA machinery, Cfd1–Nbp35 [37, 38]. Next, the cluster is transferred through the pathway molecules to the cytosolic apoproteins for their maturation (Fig. 2).

## Fe–S clusters in mammalian cells

As noted by the fact that the assembly involved in the biogenesis of Fe–S clusters is highly conserved throughout evolution, mammalian Fe–S cluster biogenesis follows a path that resembles both the yeast and bacterial pathways. The bacterial classical system is mainly regulated by the *Isc* gene [39] and homologues of the proteins encoded by this gene can be found in mammals. In this section, we will focus on the mammalian biogenesis machinery as a basis to show its involvement in several diseases such as neurodegenerative disorders.

The process of the de novo synthesis of Fe–S clusters takes place in mitochondria and is driven by the ISC machinery. Furthermore, it has been suggested that Fe–S biogenesis can also take place in the cytosol and the nucleus [40–42]. The synthesis starts with the delivery of both the sulfur and iron ions onto the scaffold protein ISCU [43]. The sulfur is provided by a cysteine desulfurase (NFS1) which removes and delivers it from l-cysteine onto ISCU. This delivery process is driven by the formation of transient persulfide on the active cysteine of NFS1, which forms a complex with ISD11 (also known as LYRM4) and the mitochondrial acyl carrier protein (ACP) [44]. The importance of the NFS1 and ISD11 has been shown by the inefficient maturation of Fe–S proteins in HeLa cells with depleted NFS1 and the accumulation of ferric iron and inactivation of aconitase in cells with downregulated ISD11, respectively [45, 46]. An allosteric regulation role for the sulfur transfer onto the ISCU–NFS1–ISD11–ACP complex has been suggested for the mammalian frataxin (FXN) [47, 48]. The iron source for the cluster assembly is not known yet; however, FXN has also been proposed as one of the iron entry regulators during the [4Fe–4S] cluster formation [49]. It is interesting to note that unlike the mammalian FXN, its bacterial homologue CyaY exerts a negative regulatory effect on the generation of Fe–S instead of its activation role present in mammals [50]. The mechanisms and players for the insertion of Fe into the complex need to be elucidated, but it has been suggested that Fe is required for the sulfur to be delivered form NFS1 [51]. Electrons are required for the generation of the [2Fe–2S] cluster and they are likely transferred from NAD(P)H to ferredoxin reductase (FDXR) and onto ferredoxin (FDX) [52].

Following its formation by the ISC machinery, the Fe–S cluster needs to be transferred to recipient apoproteins. Although little is known about this process in mammals, evidence based on yeast and bacteria suggests that the chaperone HSPA9 (HscA in bacteria) binds to the co-chaperone HSC20 (HscB in bacteria) and targets the ISCU complex for the release of the [2Fe–2S] cluster, likely mediated by a conformational change in ISCU that favours the release [53]. It has been proposed that the cluster can be released to glutaredoxin 5 (GLRX5), as the yeast Ssq1 has been shown to interact with GLRX5 to facilitate the transfer of the cluster [54]. This concept is supported by the discovery of [2Fe–2S] clusters buried in human GLRX5 revealed by its crystal structure [55]. The complex involved in late maturation of [4Fe–4S] clusters is composed of the human proteins ISCA1, ISCA2 and IBA57. This complex does not interact directly with the initial ISC machinery. The importance of each component was shown by decreased activity of aconitase, lipoic acid synthase, and the complex I of the respiratory chain in HeLa cells, where the three proteins were downregulated [56]. Finally, the trafficking from the ISCA1–ISCA2–IBA57 complex to apoproteins is facilitated by NFU1. The bacterial homologue Nfu1 interacts with the [4Fe–4S] cluster machinery and target proteins, suggesting a similar role for the human protein [57]. Furthermore, the Bol1 and Bol3 targeting factors have been involved in the maturation of a specific set of [4Fe–4S] proteins [57, 58].

The mechanism of the assembly, trafficking, and maturation of Fe–S clusters has been mainly described by the work made with the homologous proteins in both bacteria and yeast, but the relevance of most of these components in human physiology is shown by their relation to various diseases. In the following sections, we will discuss several diseases that have been linked to alterations in components involved in Fe–S cluster biogenesis.

## Is there a link between mitochondria biogenesis components and Fe–S biogenesis?

The mitochondria import pathways are critical for mitochondria biogenesis, whilst the Fe–S cluster biogenesis is considered as one of the most essential functions of mitochondria. There are some fragmented studies that suggested a link between these two important processes, but whether this is true remains unclear. Specifically, the IMS oxidoreductase Mia40, one of the two key components for the MIA pathway responsible for targeting many intermembrane space proteins, has been shown (both in vivo and in vitro) to bind to iron/sulfur clusters, in addition to its well-established role in mitochondria biogenesis [59, 60]. Two monomers can coordinate the cluster through the catalytic CPC motif. However, it remains unclear whether the presence of Fe–S on Mia40 has any relevance for its import function. In mammalian cells deletion of Mia40 is associated with increased iron levels in mitochondria, but again whether this is a direct effect on Fe–S cluster availability/transport or an indirect effect of reduced import of some other protein(s) that are Mia40-dependent is not known.

Another link of the two processes is through the CIA component, Dre2, that has been shown to localize both in the cytosol and in mitochondria [61–63]. Dre2 interacts with Mia40 independently of the presence of its Fe–S clusters. This interaction results in the introduction of two disulfide bonds in the Dre2 structure. However, it was later demonstrated that the localization of Dre2 is difficult to prove unambiguously as this protein associates tightly with the outer membrane of mitochondria and resists proteolytic hydrolysis [64]. Even though the role of Dre2 in the cytosol as part of the CIA machinery is understood, a putative role for a mitochondria-associated sub-population has not been determined yet. One could speculate that the mitochondrial association may be triggered by specific determinants potentially assisting the delivery of the iron–sulfur clusters from the matrix into the cytosol.

Most of the mitochondrial biogenesis pathways are well conserved. One exception is the trypanosomatids, a family of protozoan parasites. It has been shown that oxidoreductase Mia40 is absent from this organism, while the Erv1 homologue seems to take over the entire function of the Mia/Erv1 pathway [65, 66]. It is not clear how this system in *T. brucei* can operate mechanistically. The investigation of the Fe–S cluster export pathway in this organism will be very exciting.

Intriguingly, the initial results that linked lower levels of Erv1 in a yeast temperature mutant to a role of this protein in Fe–S cluster were later disputed by another study. This showed that glutathione levels in this strain were surprisingly lower and it is the effect of the levels of glutathione, the apparent cause for the observed defect in Fe–S cluster biogenesis [67]. These authors went to a great extent to investigate different strains and conditions relevant to both Erv1 and Mia40, and concluded that there is no link of the levels of iron to Erv1 or Mia40.

Another case of an iron–sulfur cluster protein that has been shown to have mitochondrial association is mitoNEET [68]. Specifically, this protein is anchored to the outer membrane facing the cytosol; and even though it has a zinc finger domain, it binds to iron, specifically [2Fe–2S] clusters. Many studies focus on this protein, as its overexpression is linked with various effects, such as enhanced lipid uptake, reduced membrane potential, less ROS production, and inhibition of iron transport from the cytosol in mitochondria [69]. All the above point to a role of mitoNEET as an important mitochondrial regulator and an interesting potential target for therapies. MitoNEET has already been used as a target of the insulin-sensitizing thiazolidinedione diabetes drugs [70].

## The case of oxymonad *Monocercomonoides* sp.

Mitochondria are considered in general indispensable for viability. Supporting this concept, in the past, there have been reports of organisms surviving in low oxygen environments with a reduced form of this organelle. Recently, however, a very interesting study reported the first case of eukaryotic organism completely devoid of mitochondria, the oxymonad *Monocercomonoides* sp. [71]. It appears that the absence of mitochondria in this case is not an ancestral characteristic, but instead a secondary loss incident. The data are based mainly in phylogenetic analysis and genome searches for known genes, normally selected as markers in mitochondrial studies, for example membrane translocases. The searches proved unsuccessful as no such sequences are present in the *Monocercomonoides* sp. genome. Regarding the Fe–S cluster biosynthesis pathways, however, this organism contains a cytosolic sulfur mobilization system (SUF), that has been described in bacteria, instead of the mitochondrial iron–sulfur cluster assembly pathways (ISC). In addition to the SUF, the group identified other essential genes for glycolytic proteins including enzymes for anaerobic glycolysis, indicating that the mitochondrial absence is solid. Thus, the existence of *Monocercomonoides* sp. highlights a unique exception to the concept that mitochondria are essential for viability in all eukaryotes.

## Iron sensing and regulation

Well-studied and known reactions that utilise Fe–S clusters include the sulfur donors in biosynthesis, the mitochondrial electron transport chain reactions, catalysis by aconitase, etc. These reactions have been linked with human diseases via elevated iron levels, mutations in enzymes, or DNA damage.

As mentioned before, even though iron is vital for the cell processes, excess amounts can lead to oxidative damage and toxicity. Cells have adapted to address to such a challenge, by developing specific mechanisms to strategically regulate iron intake, storage, and utilization according to the variations in cell environment.

Iron regulatory proteins (IRPs) are responsible for controlling the translation of genes involved in those specific mechanisms by binding to non-coding sequences of the corresponding mRNAs, known as iron-responsive elements (IREs). This mechanism depends on the cell iron conditions, in addition to structural features of the enzyme [72]. Thus, in the presence of high iron levels, IRP1 binds a cubic iron–sulfur cluster and function as a cytosolic aconitase. There are many dedicated studies on IRP1, their structural features, interaction with hypoxia inducing factors that are covered in other reviews [73, 74].

Another example is in *Saccharomyces cerevisiae*, where the iron metabolism is regulated by the transcription factors Aft1/Aft2 and Yap5, in accordance to the available iron levels [24, 36]. The first two are implicated with the activation of genes when the iron levels are low, while Yap5 is involved when the iron levels are extremely high. In particular, these proteins receive a signal from the mitochondrial ISC, in the form of a cluster, and with the help of the monothiol glutaredoxins Grx3 and Grx4, they regulate their transcriptional function [35, 75–77]. All genes associated with iron sensing and regulation include a specific DNA sequence in their promoter, the iron regulatory element (known as IRE), that is recognised by the transcription factors.

There are also many indications that tripeptide glutathione (GSH) plays an essential role in cellular iron metabolism [78, 79]. Defects in glutathione biosynthesis can lead to accumulation of iron in mitochondria [80, 81].

## Diseases associated with iron–sulfur clusters

The involvement of iron–sulfur clusters in the vast majority of the cell processes makes it clear that many diseases are linked with malfunctions or mutations in pathways that include them. Below, we mention some of the many known and studied diseases associated with Fe–S pathways, also summarised in Table 1.

**Table 1:** Diseases caused by alterations in the Fe–S cluster biogenesis machinery

Disease	Yeast protein	Human protein	Function	Tissue affected	References
Friedreich’s ataxia	Yfh1	Frataxin	Frataxin (FXN) is involved in the regulation of the early steps of Fe–S cluster assembly	Dorsal root ganglia, heart	Bradley et al. [85], Filla et al. [83]
ISCU and FDX2 myopathies	Isu1 and Isu2/Yah1	ISCU/FDX2	ISCU is the main scaffold protein for assembly of the cluster	Heart and skeletal muscle	Kollberg and Holme [93], Mochel et al. [91], Spiegel et al. [94]
			FDX2 is proposed as electron donor for the cluster biogenesis		
Infantile complex II/III deficiency	Nfs1	NFS1	NFS1 is the cysteine desulfurase responsible for the supply of sulfur	Multisystem organ failure	Farhan et al. [95]
Respiratory chain complexes deficiency	Isd11	ISD11 (LYRM4)	ISD11 is part of the core complex participating in the stabilisation of NSF1	Skeletal muscle and liver	Lim et al. [96]
X-linked sideroblastic anemia and ataxia	Atm1	ABCB7	ABCB7 is part of the export machinery	Central nervous system	Allikmets et al. [98], Bekri et al. [99]
Sideroblastic anemia or varian non-ketonic hyperglycinemia	Grx5	GLRX5	GLRX5 is suggested to be involved in the targeting of preformed clusters	Red blood cells, spleen and liver	Ye et al. [100]
Mitochondrial encephalopathy	Ind1	IND1 (also known as NUBPL)	IND1 is involved the transfer of the cluster to complex I of respiratory chain	Skeletal muscle and central nervous system	Calvo et al. [101]
Multiple mitochondrial dysfunctions syndromes	Nfu1, Aim1 and Iba57	NFU1, BOLA3 and IBA57	NFU1 and BOLA3 are involved in the cluster delivery to specific proteins IBA57 is believed to act in the assembly of 4Fe-4S cluster in specific recipients	NFU1 disorder is not tissue-specific BOLA3 affects primarily the central nervous system but has been reported in multisystem organ failure IBA57 affects skeletal muscle and central nervous system	Baker et al. [105], Haack et al. [103], Seyda et al. [104]

### Friedreich’s ataxia

Friedreich’s ataxia (FRDA) is the most common disease associated with dysfunction of Fe–S biogenesis. It is an autosomal recessive neurodegenerative disease with an incidence of 1/50,000 in Caucasian population [82]. FRDA is caused by a homozygous guanine–adenine–adenine (GAA) repeat expansion within the first intron of the frataxin gene (*FXN*) located on the long arm of chromosome 9 [83]. The main symptoms have been associated with FRDA dysarthria (a motor speech disorder), scoliosis, muscle weakness, and loss of position sense. In addition, FRDA can lead to diabetes and cardiomyopathy.

As mentioned before, frataxin is involved in Fe–S clusters biogenesis. Thus, alterations linked to iron metabolism are present in FRDA. The pathophysiology of FRDA comprises deficit of aconitase and respiratory chain complexes, presence of oxidative damage markers in blood and urine, and intracellular iron accumulation [82, 84–86]. Currently, no successful treatment is available for FRDA. One main reason for this is the lack of understood detailed understanding of mechanisms of Fe–S cluster biogenesis and appropriate disease models. However, some treatments involving iron chelators like deferiprone are already in clinical trials and are listed in the Friedreich’s Ataxia Research Alliance; and others are still being developed and tested in animals (i.e., gene therapy targeting the FXN gene) [87, 88].

### ISCU and FDX2 myopathies

Myopathy is a disorder of skeletal muscles with the presence of impairment of muscle fibers. The ISCU myopathy is an autosomal recessive disorder characterised by exercise intolerance that leads to increased lactate and pyruvate concentrations and it was first identified in Sweden [89]. This disorder is caused by severely reduced levels of ISCU protein in individuals that share a point mutation in the fourth intron of the *ISCU* gene that causes a premature stop codon. This mutation amplifies a polypyrimidine tract (tcttt**g** to tcttt**c**), which is normally a weak splice acceptor. As a consequence of this, the strengthened site allows the inclusion of an aberrant exon into the transcript [90]. The reduced levels of a functional ISCU protein lead to Fe–S clusters biogenesis and Fe homeostasis alterations in samples from patients [91]. A mutation of a conserved glycine residue into glutamate has been shown to interfere with the interaction of the scaffold protein with NFS1 and HSC20 [92]. The use of specific anti-sense oligonucleotide targeting the *ISCU* gene to correct the reading frame has shown promising results in fibroblast derived from patients [93].

Similar to the ISCU myopathy, mutations in the *FDX1L* gene which disrupts the initiation translation site of the FDX2 protein caused proximal muscle myopathy associated with myoglobinuria and lactic acidosis together with reduced activity of aconitase and affected complex II [94].

### Infantile complex II/III deficiency

A lethal autosomal recessive disease caused by a mutation that leads to substitution of arginine into glutamine in the cysteine desulfurase, NFS1, has been identified. It is characterised by lactic acidosis, muscle deficiencies in respiratory complex II and III that have as consequence multisystem organ failure [95]. Little is known about this disorder as just three patients have been reported so far, but its lethality as well as the strong impairment of both cytosolic and mitochondrial Fe–S proteins in NFS1 knocked down HeLa cells stress the importance of the activity of this protein.

### Respiratory chain complexes’ deficiency

A homozygous mutation in *LYRM4*, the gene encoding for ISD11, was found in two patients with decreased oxidative phosphorylation. The mutation was identified in a patient with deficiency of complex I, II, and III in muscle and liver by massive parallel sequencing (MitoExome sequencing) [96]. The same mutation was identified in another affected patient who presented additional complex IV deficiency and who died while neonate. The differences between the outcomes of both patients are suggested to be due to the availability of cysteine in the new-born period. Furthermore, in vitro experiments showed no desulfurase activity of NFS1 when expressed together with mutant ISD11, reflecting the importance role of this protein for the stabilisation of the desulfurase [96].

### X-linked sideroblastic anemia and ataxia

The ATP-binding cassette, sub-family B, member 7 (ABCB7) is a membrane associated protein located in mitochondria. ABCB7 is a transporter present in the inner membrane of mitochondria and its depletion in HeLa cells showed Fe accumulation within mitochondria [97]. Mutations in the *ABCB7* gene cause a recessive disorder, namely X-linked sideroblastic anemia and ataxia (XLSA/A) [98]. XLSA/A is an early onset disease characterised by a blood disorder in which erythrocytes do not produce enough haemoglobin (sideroblastic anemia) and slow-progressive movement problems (slow-progressive ataxia) [99]. The mutations on XLSA/A patients are in the transmembrane domain, but still, complete understanding of ABCB7 function is needed to fully characterise this disorder and can develop a possible cure.

### Sideroblastic anemia or variant non-ketonic hyperglycinemia and iron overload

Sideroblastic anemias are a group of heterogeneous disorders that share common features like mitochondrial iron overload, high numbers of ringed sideroblasts, and affected erythropoiesis. Among the different types of sideroblastic anemia, one version is caused by a large deletion on the *GLRX5* gene. This mutation in intron 1 leads to complete loss of GLRX5 protein function and the subsequent impairment of Fe–S biogenesis together with the activation of the iron-responsive element (IRE)-binding activity of iron regulatory protein 1 (IRP1) [100].

### Multiple mitochondrial dysfunctions syndromes

Multiple mitochondrial dysfunctions syndromes (MMDS) are due to mutations in proteins involved in biogenesis of Fe–S clusters and are characterised by mitochondria affected at multiple levels. MMDS 1 is caused by mutations in affecting NFU1 and was first identified in three siblings of Mexican origins, while MMDS 2 is the result of a mutation that leads to premature stop codon in the *BOLA3* gene causing severe epileptic encephalopathy and elevated lactate and glycine levels. Finally, MMDS 3 is caused by a homozygous mutation that diminishes the activity of IBA57 which leads to defects in mitochondrial respiratory complexes I and II [102–105].

## Conclusion

Despite the rapid progress in the Fe–S field, there are still many unanswered questions regarding the interactions between the individual proteins, the formation of complexes, the identity or even the presence of an X-S exported material from the mitochondria, and the delivery of the clusters into the recipient apoproteins. Research in this field will help to understand the determinants of many diseases associated with iron cluster protein pathways. Furthermore, the development of appropriate cell and mouse models for these diseases is crucial to further investigate potential therapeutic strategies.

## Abbreviations

Aft: Activator of ferrous transport
Atm1: ATP-binding cassette (ABC) transporter
Cfd1: Complement factor D
CIA: Cytosolic iron–sulfur cluster assembly
Erv1: Essential for respiration and vegetative growth 1
GFER: Growth factor, augmenter of liver regeneration
Grx/GLRX: Glutaredoxin
GSH: Reduced glutathione
GSSG: Oxidised glutathione
HSP9: Heat shock protein 9
HSC20: Heat shock cognate protein 20
IRP: Iron regulatory proteins
IRES: Iron-responsive elements
ISC: Iron–sulfur cluster
Isu1: Iron–sulfur cluster assembly scaffold protein
Jac1: J-type accessory chaperone 1
Nbp35/NUBP1: Nucleotide-binding protein
Rad3: TFIIH/NER complex ATP-dependent 5′–3′ DNA helicase subunit RAD3
Ssq1: Stress-seventy sub-family Q 1
SUF: Sulfur mobilization system
XPD: Xeroderma pigmentosum group D helicase
Yap5: Yeast AP-5
